# Risk Factors for Chronic Stress in Sows Housed in Groups, and Associated Risks of Prenatal Stress in Their Offspring

**DOI:** 10.3389/fvets.2022.883154

**Published:** 2022-04-12

**Authors:** Martyna Ewa Lagoda, Joanna Marchewka, Keelin O'Driscoll, Laura Ann Boyle

**Affiliations:** ^1^Pig Development Department, Teagasc, Animal and Grassland Research and Innovation Centre, Fermoy, Ireland; ^2^Department of Animal Behaviour, Institute of Genetics and Animal Biotechnology, Polish Academy of Sciences, Jastrzebiec, Poland

**Keywords:** swine, piglet, gestation, prenatal, chronic, stress, welfare, risk

## Abstract

Chronic stress has a detrimental effect on sow welfare and productivity, as well as on the welfare and resilience of their piglets, mediated prenatally. Despite this, the specific risk factors for chronic stress in pregnant sows are understudied. Group-housed pregnant sows continuously face numerous challenges associated with aspects of the physical (group type and size, flooring, feeding system) and social (stocking density, mixing strategy) environment. There are many well-known potent stressors for pigs that likely contribute to chronic, physiological stress, including overcrowding, hot temperatures, feed restriction, inability to forage, uncomfortable floors, and poor handling. Some of these stressors also contribute to the development of production diseases such as lameness, which in turn are also likely causes of chronic stress because of the associated pain and difficulty accessing resources. The aim of this review is to discuss potential risk factors for chronic stress in pregnant sows such as space allowance, group size and type (stable/dynamic), feeding level, lameness, pen design, feed system, enrichment and rooting material, floor type, the quality of stockmanship, environmental conditions, and individual sow factors. The mechanisms of action of both chronic and prenatal stress, as well as the effects of the latter on offspring are also discussed. Gaps in existing research and recommendations for future work are outlined.

## Introduction

Implementation of Couuncil Directive 2001/88/EC, 2001 saw the transition from confinement of sows in individual stalls during gestation to group housing in the European Union. This is a trend mirrored in pig producing countries worldwide [e.g., Proposition 12 in the United States (1, 2)]. Group housing systems are considered more welfare-friendly as they allow sows a greater degree of freedom of movement and an opportunity for social interactions (3) compared to confinement in stalls. However, group housing comes at a price of other challenges, including sustained aggression among sows due to the competition for limited resources, social conflicts caused by continuous re-mixing, subordination/isolation of individuals, as well as suboptimal physical environments, all of which could lead to long-term (chronic) stress (4–6).

There are numerous studies investigating the types and physiological consequences of acute stressors that sows experience (7–9). One of the most commonly studied acute stressors is mixing of unfamiliar individuals, which results in fighting to establish a dominance hierarchy. This is associated with high levels of stress, manifested as elevated heart rate, plasma catecholamines (10), and cortisol levels. In fact, mixing is also a major acute stressor for weaner, grower, and finisher pigs (11, 12), with studies showing evidence of profound physiological and behavioural changes following mixing (11, 13). Overall, this confirms that mixing is a highly stressful event (11). Other examples of acute stressors include transport, social isolation, and physical restraint (7–9). For instance, Bradshaw et al. (7) and Soler et al. (9) found higher salivary cortisol levels in pigs shortly after they experienced rough transport conditions. Higher serum amyloid A and cortisol concentrations were shown in pigs isolated for short periods of time (9). In addition, increased cortisol and serum amyloid A concentrations were also recorded in pigs subjected to physical restraint (8).

While most work on stress in pigs focuses on weaner and finisher pigs, generally, there is limited knowledge on stress in pregnant sows (other than while in the farrowing crate), with even less information on chronic stress (14–17). This is despite the fact that some acute stressors could contribute to chronic stress if experienced repeatedly, such as repeated remixing of unfamiliar individuals (18), or competition for limited resources and the associated aggression (4). Therefore, it is evident that chronic stress could be experienced by sows to a greater extent, as the additive negative effects of repeated acute stressors were not previously considered as contributing factors (19–21). This is a major cause for concern as stress experienced by the mother throughout gestation has negative effects not only for the sow herself, but also on foetal development, with the potential to persist into the offspring's adulthood (22). This is known as prenatal stress, and could have negative implications for offspring resilience to disease, welfare challenges, productivity and performance.

It is possible to make inferences about levels of chronic stress experienced by animals based on performance, behavioural, and physiological parameters (23). For instance, impaired reproductive performance can be a symptom of chronic stress, as energy resources are redirected away from maintenance and developmental processes, including pregnancy (24, 25), and diverted towards processes aimed at ensuring survival (26). Likewise, stereotypic behaviours can become established in situations where animals are chronically stressed (6). Other behaviours indicative of chronic stress in pigs include abnormal levels of vocalisations, urination/defecation, and inactivity (6, 27). Chronic stress also leads to immunosuppression, which in turn results in higher disease incidence (28, 29). Other physiological indicators of chronic stress include increased levels of cortisol [e.g., in hair (30)], and altered patterns of cortisol concentrations in faeces, blood plasma, and saliva (31).

The medium in which cortisol is measured can have an effect on the resulting concentration. Hence, the choice of medium must be considered carefully, to ensure it is appropriate for the specific type of stress under investigation, i.e., acute or chronic. For instance, there is increasing focus on measurement of cortisol in hair as an indicator of chronic stress due to the long-term accumulation of cortisol within the growing hair shaft (32–34). While hair collection is a non-invasive procedure with potential to give insight into stress levels over weeks or months (30, 32), there are also many confounding factors (collection site, hair colour, age, sex, stage of gestation, cleanliness) that affect cortisol levels in hair, and which must therefore be controlled for when using this method to determine chronic stress levels (30, 32, 33). On the other hand, cortisol levels in saliva, blood plasma, urine or faeces are “point samples” strongly influenced by time of day (circadian rhythm pattern), food intake and environmental disturbances [including stress associated with the blood sampling procedure in particular; (6, 32)]. As a result, such mediums are mainly used to quantify short-term stress levels (6, 32). Saliva and blood plasma capture stress levels experienced over minutes, while urine and faeces capture slightly longer periods which might span days (6, 32, 35). However, it is still possible to use such mediums to quantify chronic stress levels, provided they are measured consistently over time [Davenport et al. (32)]. Doing so allows patterns to be identified, which in turn can reveal deviations from the norm, indicative of chronic stress (31, 32, 34).

Although there are numerous indicators that allow researchers to make inferences about levels of chronic stress, there are no confirmed risk factors within the sow's environment. Given the postulated negative effect of chronic stress on both the sow and her offspring (21, 22), there is an urgent need for additional research to identify the potential risk factors for chronic stress experienced by pregnant sows. It is also necessary to ascertain the potential for such factors to contribute to prenatal stress and associated reduced resilience in the offspring of chronically stressed sows. There are numerous aspects of the physical and social environment to which sows are continuously subjected throughout gestation, which could act as potential risk factors for chronic stress. These include space allowance (36, 37), group size and type [stable/dynamic; (38)], feeding level (39, 40), lameness (30, 41, 42), pen design (4, 43), feed system (23, 44), enrichment and rooting material (1, 45), floor type (46–48), quality of stockmanship (49, 50), environmental conditions (51), and certain individual sow factors (52, 53). The aim of this review is to discuss such factors in terms of their ability to induce chronic stress in sows. The mechanisms of action of both chronic and prenatal stress, as well as the effects of the latter on offspring are also discussed. Gaps in existing research and recommendations for future work are outlined.

## Chronic and Prenatal Stress–Mechanisms of Action

### Mechanisms Underlying Chronic Stress

Stress is a phenomenon defined as a “non-specific response of the body to any demand” (54, 55). Stressors which drive this response are of variable nature, and can be both physical and psychological (54, 56). While stressors act on many different regions of the nervous system to induce appropriate responses (54), the most prominent features of the stress response involve the activation of the autonomic nervous system and the hypothalamo-pituitary-adrenocortical (HPA) axis (54, 57, 58). At its most basic, this involves the synthesis of cortisol (glucocorticoid stress hormone) by the adrenal cortex in response to adrenocorticotropic hormone (ACTH) (57). This in turn has numerous knock on effects on a range of internal processes (57, 58). Moreover, these effects differ depending on whether the stressor is acute (lasting minutes or hours), persists chronically (for days, weeks, or even months), or whether the organism is repeatedly exposed to acute stressors [chronic intermittent stress; (18)], as well as depending on the severity of the stressor (57, 58). In fact, the results from existing research on HPA axis activity are conflicting (59). Some authors argue that HPA axis activation does not always reflect stressful conditions, as it is known that it can be either upregulated or downregulated in response to chronic stress, depending on the situation and the individual involved (34, 59, 60). Others suggest that a generalised endocrine profile of a chronically stressed animal does not exist, as there is so much variation in the stress response of individual animals (61). Furthermore, while accounting for individual differences in animal biology was overlooked due to the historical focus on group as the experimental unit, many still highlight the importance of considering individual animals in the design of experiments (62, 63). Not all animals in the group respond in the same way to stressors, or indeed to their overall environment (63). This is highlighted by research investigating animal personalities and coping styles, defined as “alternative response patterns in reaction to a stressor” (63). For instance, animals that respond to a stressor with high levels of offensive, aggressive behaviour are said to adopt a proactive (active) coping style, while animals responding with low levels of offensive aggressive behaviour are said to adopt a reactive (passive) coping style (63, 64). Moreover, the resulting variation in responses could differentially impact offspring. Following on from this, Herman et al. (31) state that chronic stress-induced, protracted activation of the HPA axis takes many forms, including prolonged basal hypersecretion of glucocorticoids, sensitised stress responses, and even adrenal exhaustion, and that this can depend on the duration of the stressor, as well as its intensity, frequency and modality (31). Thus, caution must be exercised when interpreting chronic stress levels based on HPA axis activity patterns alone (59, 60).

### Effects of Chronic Stress

Nonetheless, evidence exists of the negative effects of prolonged activation of the HPA axis during the experience of chronic stress. This includes immunosuppression by cortisol (predominant mediator of stress in situations whereby stressful stimuli are prolonged), resulting in increased susceptibility to disease, due to decreased numbers of lymphocytes, cytokines and immunoglobulins in the blood of chronically stressed animals (6). This can in turn mean that energy resources in stressed animals are redirected away from maintenance and developmental processes, including pregnancy (24, 25), impairing reproductive performance (28, 29). Chronic stress can also impair reproductive performance by inhibiting the release of both luteinizing hormone and progesterone (6).

Chronically stressed animals can also have an enhanced or a diminished response to acute stressors (65–69). In terms of effects on behaviour, frustration associated with an animal's inability to cope with a challenge, or having no control over its immediate environment/social situations can lead to chronic stress, which in turn can stimulate the development of stereotypic behaviours (6). Research into the functional significance of stereotypic behaviours suggests that their performance acts to reduce the stress associated with the situation which initially caused it [i.e., acting as a stress coping mechanism; (70)]. This is supported by evidence from studies demonstrating reduced heart rates in stereotyping equines (71, 72), increased plasma cortisol concentration in horses prevented from stereotyping (72, 73), and a decrease in faecal corticoids in stereotyping macaques (74). Consequently, while stereotypic behaviour is indicative of suboptimal environments and the chronic stress associated with them [either past or present; (75)], it may not be an accurate indicator of current physiological stress as measured by heart rate or glucocorticoid levels (72). On the other hand, neurotransmitters such as serotonin are implicated in the pathology underlying stereotypic behaviour, with lower basal levels found in stereotyping animals (72). Therefore, measuring serotonin levels could be a better method of assessing the pathological nature of stereotypic behaviours (72).

### Prenatal Stress–Mechanism of Transfer to the Offspring

Based on research into the effects of acute prenatal stress events on offspring, it is now known that prenatal stress is mostly hormonally mediated in many mammalian species, including guinea pigs, mice, rats, and swine (14, 76). Moreover, chronic maternal stress experienced during gestation can also cause chemical changes in the mother's body, which in turn can lead to increases in cortisol levels, and associated negative consequences for the developing offspring [guinea pig (14); mouse, rat, swine (70)]. Depending on the species, there are different types of placenta, with structural differences (77). Ultimately, the placenta acts as an interface between the mother and foetus (78). While the placenta forms a barrier to many chemicals, some, including glucocorticoids, will still pass through and have an effect on the developing foetus (15). For instance, Welberg et al. (69) demonstrated that acute stress can upregulate the chemical activity of placental 11β-hydroxysteroid dehydrogenase type 2 (component of the foetal-placental barrier to maternal corticosteroids) in rats, thus protecting the foetus against elevated maternal cortisol levels. However, under chronic stress conditions, the capacity to upregulate placental 11β-hydroxysteroid dehydrogenase type 2 activity in the face of an acute stressor is reduced by 90%. Thus, maternal exposure to chronic stress diminishes the placental capacity to protect the foetus from elevated maternal cortisol levels, with negative effects on the developing offspring (69). Maternal glucocorticoids can activate the foetal HPA axis and alter its development, with consequences for offspring stress coping mechanisms later in life [demonstrated in primates, guinea pigs, sheep, cattle, goats, pigs, rats and mice (15)]. For instance, prenatal stress dysregulates functionality of the HPA axis in species of monkeys and rodents in a way that leads to decreased feedback inhibition of corticotropin releasing hormone, causing prolonged elevation of circulating glucocorticoids in response to stress in later life (79).

Besides glucocorticoids, other maternal circulating hormones and chemicals such as catecholamines, also mediate prenatal stress (80). For instance, Kapoor et al. (15) found that increased maternal catecholamine concentrations in rats resulted in constriction of placental blood vessels, causing foetal hypoxia. This in turn caused the activation and reprogramming of the foetal sympathetic system, again resulting in altered offspring physiological responses to stress later in life (15).

More recently, the maternal vaginal microbiome was proposed as another potential mediator of prenatal stress (81). Vaginal microbiota harvested and transplanted from chronically stressed mouse dams into their naïve offspring delivered by caesarean section had effects which resembled those seen in naturally prenatally stressed offspring. These effects included changes in the foetal intestinal transcriptome and in hypothalamic gene expression (81).

#### Prenatal Stress in Swine Offspring

The stage of gestation during which a stressor occurs is also an important factor to consider (82–86), because various systems of the developing embryo/foetus are vulnerable to stress at different times throughout prenatal development (83). For example, early gestation (day 10 to day 17) is a critical period for pig embryo establishment and development. Couret et al. (85) showed that early gestational stress in the form of a social stressor led to an increased adrenal weight, while late gestational stress resulted in an increased proliferation index of blood cells in sow offspring. Omtvedt et al. (87) demonstrated differential effects of heat stress experienced by pregnant sows in early, mid and late gestation on the prenatal development of offspring. For example, heat stress experienced in early gestation interfered with embryo development and implantation (87). Likewise, Lucy et al. (88) showed that heat stress increased embryo mortality during early gestation, but led to a higher number of stillborn piglets if experienced later in gestation. Mixing is also a major stressor for sows, and also an example of a stressor with different effects on prenatal development depending on the stage of gestation during which it occurs (89). Mixing in early gestation generates sufficient prenatal stress to increase embryonic mortality and decrease the future litter size, in contrast to mixing during the fourth week of gestation (90). Lagoda et al. (91) demonstrated that mixing in early gestation can generate stress which persists chronically, with detrimental effects on reproductive performance in subsequent parities. It is thus possible that stress associated with early mixing, acting prenatally, could have long-term, carry-over effects on the affected offspring that survive to birth. Overall, it is clear that irrespective of the type of stressor which causes the maternal stress response, experience of prenatal stress in early gestation is especially detrimental to the developing offspring.

## Risk Factors for Chronic Stress in Pregnant Sows

### Space Allowance

Space allowance encompasses the physical space which the animal occupies and needs to change posture, stand up or lie down, as well as the additional space it needs to exercise and maintain muscle tone (92). When investigating the effects of space allowance on sow stress levels, certain confounding factors must be considered. For instance, both quality and quantity of the available space are important factors that can influence the stress levels. Often, factors such as the amount of “free” shared space available to group-housed sows, or whether extra space is required by larger sows, are not considered (93). Moreover, some authors advise caution when interpreting the effects of different space allowances, as group size can act as a confounder (93), while others show few or no interactions between group size and space allowance (94).

The feeding system in use can also impact the space available to the animals (1). Individual feeding stalls take up more space than a single electronic sow feeder (ESF), and thus the stocking density of the sows must be considered in relation to the actual space allowance available to each sow (1).

Animals also require adequate space for social interactions, such as establishment of a dominance hierarchy, avoidance of aggression, and performance of natural behaviours for which they are highly motivated (92). As such, restriction of space is associated with chronic stress in all species [i.e., fish: Sundh et al. (95); birds: Selvam et al. (96); cattle: Schubach et al. (97)]. Indeed, the behavioural diversity of sows housed at lower space allowances can be curtailed (98), and inability to perform a full behavioural repertoire is a source of frustration and stress for animals (6). Following on from this, inadequate space allowance can lead to overcrowded conditions, exacerbating agonistic interactions between pen mates (99, 100), which leads to elevated cortisol levels, indicative of stress (38).

It is also possible that adequate space allowance is crucial to the animal's ability to maintain personal space. For instance, Greenwood et al. (101) demonstrated benefits of increased space allowance at mixing, especially in the case of low ranking sows. In that study, sows in the highest space allowance treatment also had the highest cortisol concentrations. The authors explain this to be a consequence of increased levels of activity within this treatment, rather than a consequence of increased aggression or stress (101). On the other hand, Hemsworth et al. (37) and Barnett et al. (36) confirmed negative effects of reduced space allowance as indicated by chronically elevated cortisol levels in sows housed at a low space allowance. Lower space allowance was also associated with a lower percentage of gilts in oestrus, suggesting an impairment of sexual behaviour and reproductive performance at lower space allowances (37). Not meeting space allowance requirements therefore exerts stress on the animals, which can potentially act as a risk factor for chronic stress.

### Group Size and Type (Stable/Dynamic)

Elucidating the effects of group size on sow stress levels is difficult because of confounding factors influencing levels of aggression in a group. These include the effects of the group type (dynamic vs. static) or space allowance, given that the optimal space allowance for sows at times of high aggression is unknown (94). The effect that group type has on aggression could mask the effect of group size on stress levels in sows. For example, in dynamic groups, the addition of new individuals continuously disrupts the dominance hierarchy, resulting in an increased intensity of fighting to establish the rank order (102, 103). This is in contrast to static groups, where the dominance hierarchy is established once, after which the intensity of fighting diminishes. Therefore, dynamic groups themselves could act as a potential risk factor for chronic stress. Moreover, following on from the constant addition of new individuals into dynamic groups, the size of dynamic groups is often larger than that of static groups, resulting in more hierarchy conflicts to resolve, and leading to higher levels of aggression (104). Consequently, sows housed in dynamic groups have higher cortisol levels compared to sows in stable groups (105).

However, such conclusions warrant a degree of caution, as it is not clear whether increased aggression levels in dynamic groups result from larger group size, or from the constant disruption of the dominance hierarchy due to the addition of new individuals (104). Most likely, it is a combination of both. However, Misra et al. (106) showed lower levels of aggression in large stable groups compared to small stable groups of finisher pigs. Therefore, it is possible that levels of aggression are also lower in large compared to small groups of pregnant sows provided the groups are stable. Unfortunately, investigations of effects of group size on aggression often test the same group sizes at different space allowances. Hence, while Hemsworth et al. (94) showed that in static groups, smaller group sizes (*n* = 10 sows) were associated with fewer injuries than in large groups (*n* = 30 or *n* = 80 sows), Taylor et al. (107) showed no effect of group size on skin injuries. Due to differences in space allowance, such results cannot be compared, and thus the effect of group size is unclear. Nonetheless, with more hierarchy conflicts to resolve in large groups, and therefore increased aggression levels, large group size is a plausible candidate risk factor for the development of chronic stress in sows.

### Feeding Level

Sows in commercial systems are feed-restricted during pregnancy to ensure optimal body condition when it comes to production of viable piglets (108, 109). The aim is to optimise reproductive performance and ensure correct timing of return to estrus after weaning (40, 110, 111). While the restricted feed ration is sufficient to meet general maintenance requirements, ensure good health and performance, and adequate maternal and embryonic tissue growth, it does not ensure satiety (112). Providing feed restricted sows with high fibre diets allows to minimise the negative effects of restrictive feeding (40, 109, 113, 114). High fibre diets including roughage materials such as straw and grass silage, or bulky materials such as beet pulp promote a feeling of satiety, and thus reduce the motivation to continue feeding, and ameliorate associated frustration and hunger (40, 112, 114).

Moreover, in the absence of roughage, feed restricted sows remain highly motivated to eat. For instance, when tested in an operant task, feed restricted pigs were highly motivated to continue feeding by accessing extra feed (112, 115). This research revealed that the restricted ration typically allocated to pigs accounted for only 60–70% of the quantity of food they were capable of eating *ad libitum* (112, 115). Thus, the motivation to feed persists, resulting in chronic hunger, frustration, and increased stress levels as indicated by elevated cortisol concentrations (39, 40), as well as increased stereotypic behaviour performance (109, 116). As a consequence, there is competition for feed resources among feed restricted sows, leading to high levels of and more intense aggression (40, 117). Several studies found associations between restricted feeding and stereotypic behaviours (109, 116), which signal increased stress levels of feed restricted sows (118). This, combined with the high motivation to continue feeding, makes for compelling evidence that feed restriction is a risk factor for chronic stress for pregnant sows.

Nevertheless, it is difficult to quantify the levels of chronic stress associated with restricted feeding regimes. In contrast to Amdi et al. (39) who found elevated cortisol levels in feed-restricted sows, certain studies showed no changes in cortisol concentrations, and thus in stress levels of feed restricted animals (109, 119, 120). Although measuring cortisol levels is the standard when it comes to quantifying stress in animals, it is also possible that cortisol may not be a suitable physiological indicator of stress associated with hunger (121). This is because corticosteroids are affected by metabolic rates, which in turn relate to the state of hunger, potentially acting as a confounder (121).

### Lameness

Lameness in sows is a common cause of reduced welfare and economic losses to pig producers (122–124). As a consequence, lame sows are often culled prematurely, reducing their longevity (122), and increasing the need to purchase replacement gilts (122).

The presence of both injuries and claw/hoof lesions, and unhygienic environments can exacerbate the development of lameness (47, 123, 125). Lameness occurs when an animal adjusts its posture or gait to minimise the experience of pain. Indeed studies investigating pain thresholds and the use of analgesics confirm that lameness is associated with pain (47, 122, 125–127). Lameness persists chronically as it often goes unnoticed due to the difficulties associated with identification of its early stages (122). Any associated long-term pain could contribute to stress both physiologically and psychologically (30, 41, 122). Physiological stress resulting from lameness is evident in studies which measured cortisol (salivary, hair), acute phase protein levels, and various salivary stress biomarker proteins (salivary α-amylase, salivary lactate dehydrogenase), with significantly higher levels of such stress related indicators in lame than non-lame animals (30, 41, 42). In addition, lameness also reduces reproductive performance (122); lame sows displayed delays in post-weaning oestrous, and had smaller litter sizes compared to non-lame sows (128).

Lameness may also contribute to psychological stress in sows, in a similar way to that reported for human patients suffering from chronic rheumatoid arthritis (129). For example, the pain and discomfort associated with lameness could render sows less successful during aggressive encounters with unfamiliar individuals when establishing a dominance hierarchy (52). This is an important component of the social behaviour of this species (52), and not being able to defend oneself from aggressors could lead to stress (93). Thus, lameness is a good candidate for a potential chronic stressor.

### Pen Design

Under commercial conditions where space is limited, pigs likely benefit from places to hide and to avoid or escape from an aggressive interaction (4, 130–132). Indeed a lack of barriers within a pen is associated with higher levels of aggression (4, 132). Barriers reduce visual contact between the aggressor and the victim (23, 133–135), reducing fear/anxiety levels in sows, as indicated by a reduction in cortisol levels (43). This suggests that the lack of barriers within a pen, particularly in the case of dynamic groups whereby dominance hierarchy is continuously disrupted, can be a risk factor for chronic stress.

### Feed System

Pigs prefer to synchronise their feeding behaviour (136), and feed restricted sows are highly motivated to access feed (39, 40). Hence competition for access to feed can cause severe aggression at feeding time (40, 136). Moreover, as feeding systems differ in the level of protection they provide to the feeding animal (23), this can affect the level of aggression that sows experience at feeding, and any associated stress (23). Protection while feeding can reduce aggression and the associated injury, stress, and disruptions to feed intake (4, 132). Feeding systems with such potential include protected ESF systems and individual full length feeding stalls, followed by troughs with barriers to separate the feeding animals. However, in the case of the latter, the level of protection depends on the length of the barriers (23). Feeding systems such as troughs without barriers, as well as floor feeding, do not provide protection during feeding time, and can thus exacerbate and prolong aggression within a group (4).

Despite providing protection at feeding and the added benefit of allowing a tailored feed allowance and diet for each individual (23), there are also certain negative aspects to protected feeding systems such as that offered by ESF systems. For example, any potential break down in the ESF system can result in sows not being fed. While the technology associated with ESF systems improved over the last decade, breakdowns are still possible and could majorly disturb the group dynamics. Another risk is that of aggression occurring as sows queue up to enter the ESF (109, 117, 137). However, this can be minimised by strategically placing the ESF away from busy pen areas and resources of interest (137, 138). It can also be ameliorated to an extent as sows establish a feeding order, with dominant sows feeding first, followed by subordinate sows (23, 139). However, due to feed restriction and the resulting chronic hunger, dominant sows continue to return to the feeder despite having eaten their daily ration (117). This results in frustration which can be expressed as vulva biting by sows waiting in the queue (109), and it also disrupts the feeding order, leading to aggression being directed towards subordinate sows still in the queue (23).

In order to avoid aggression associated with queuing to gain entry to the ESF, protected individual, free-access feeding stalls could be a useful alternative, provided that all or nearly all sows have access to a feeding stall. Indeed, Bahnsen et al. (44) showed that sows housed with protected feeding stalls had lower salivary cortisol levels compared to sows housed with an ESF system. This confirms the benefit of protected feeding stalls on sow stress levels.

### Enrichment and Rooting Material

Domestic pigs are a highly intelligent species, requiring an appropriate level of cognitive stimulation in order to maintain mental and physical wellbeing (140). In addition, domestic pigs retain a high motivation to perform exploratory behaviour, including rooting behaviour which evolved in their wild counterparts (141). The inability to perform this natural behaviour within a commercial setting due to the lack of suitable materials at which it could be directed results in frustration, and is linked to the development of damaging behaviours (142). Providing pigs with appropriate enrichment allows for cognitive stimulation, and depending on the type of substrate used, it allows the animals to fulfil their behavioural needs, including the performance of rooting behaviour (141).

The provision of enrichment also has the potential to reduce sustained levels of aggression by keeping sows occupied and less likely to get involved in aggressive behaviour [demonstrated for spent mushroom compost (143); peat (144)]. Indeed, it must be noted that this depends on the enrichment type provided. For instance, Horback et al. (145) demonstrated that while enrichment items such as ropes and wooden blocks could satisfy behavioural needs of individual sows, they could not reduce the overall levels of aggression in the pen. While its provision is not panacea when it comes to eliminating stress, there are nonetheless multiple positive effects of enrichment on sow behaviour which can reduce their stress levels (1). This explains the association between the provision of enrichment and a higher frequency of behaviours indicative of good welfare, i.e., sleeping (144). Moreover, sows housed with deep straw bedding had lower cortisol concentrations and reduced immune stimulation (lower total white blood cells) compared to sows housed without straw bedding (45). These studies support that lack of appropriate enrichment is a risk factor for the development of chronic stress in pregnant sows.

### Floor Type

Floor type can act as a risk factor for chronic stress both directly and indirectly. Its direct effect could be associated with discomfort due to lying on concrete slatted floors without bedding (23, 123). It could also directly contribute to the fear of falling and injury in instances where smooth concrete floors are slippery because of urine and faeces (46, 48, 93, 123). Additionally, indirect effects are associated with the injuries and lameness arising from certain floor types [e.g., fully slatted concrete floors (122, 123); see Lameness].

In contrast, rubber flooring can reduce the risk of claw lesion and lameness incidence, as well as improve comfort during resting and ease of changing posture (123, 146–149). In line with this, sows with access to stalls with rubber mats had lower cortisol concentrations on day 28 of gestation than sows in standard pens with concrete-floored stalls (150). In addition, sows housed on rubber floors also had improved reproductive performance (91). This finding confirms the potential of rubber flooring to reduce stress in pregnant sows (150).

On the other hand, rubber floors can become slippery, thus providing poor foothold, which may discourage sows from engaging in aggression (46, 48). In support of this, Lagoda et al. (91) showed that sows housed on rubber floors had lower skin lesion scores following mixing compared to sows on concrete slatted floors, suggesting reduced intensity of mixing aggression. Persistent slipperiness of rubber floors could also reduce aggression in the long term, as a result of fear of slipping and the consequent reluctance to engage in fights. Clearly, while slippery rubber floors may reduce the intensity of aggression, they should not be used intentionally for that purpose. Using the fear of slipping to prevent sows from fighting has its own negative connotations for welfare. Not being able to fight in order to settle dominance conflicts, as well as the constant fear of slipping would undoubtedly contribute to chronic stress (46, 151).

### Quality of Stockmanship

The quality of stockmanship is determined by the stockperson's personality, attitude, and behaviour (50, 152), and has a substantial effect on stress levels in farm animals (49, 50). Hayes et al. (153) showed the potential for positive handling to reduce fear of humans in sows, while Dokmanovic et al. (154) showed numerically lower cortisol concentrations in gently handled pigs, compared to those which were handled roughly. In contrast, Manteca and Jones (50), Hemsworth and Boivin (155) showed compromised reproductive performance resulting from rough handling and fear of humans in sows.

Despite the process of domestication and living in close proximity to humans, the initial response of farm animals to humans is still that of fear (156). This is worsened when animals are exposed to rough handling and poor quality stockmanship, and when no effort is made towards the establishment of a neutral or a positive connexion with the animals (50). This effect is exacerbated by the increasing automation of the animal production sector, which gives stock people fewer opportunities to interact with the animals in their care (50). This means that it is more difficult for animals to habituate to the presence of humans (50).

Moreover, rough handling of sows can result in a lasting aversion towards certain or all humans through classical conditioning (157). Sows handled aversively by a single stock person can learn to associate such handling with all people, thus developing a learned fear of people in general (157). Therefore, the fear of humans is not only an acute stressor which occurs at the time of handling by an abusive person. In fact, it is a lasting issue and a potential chronic stressor. This effect can be exacerbated as human handling is still inevitable at various stages of a production animal's life (50). For example, in the case of group-housed sows, handling by stock people is necessary at vaccination or when moving sows from one location to another during different stages of gestation (158). Although such handling instances are interspersed throughout gestation, for the sows with a lasting aversion and fear of humans, even intermittent handling can be extremely difficult, as well as dangerous for the stock people involved (50, 155). Fearful sows are therefore at a continuous risk of being handled adversely due to their responses to humans (158). This in turn can be associated with intense acute stress (158) occurring intermittently and contributing to chronic stress (18). Poor stockmanship is therefore a potential risk factor for chronic stress, with known detrimental consequences for sows (153).

### Environmental Conditions

Dust, gases such as ammonia, and inappropriate ambient temperature levels are just some of the environmental challenges in pig farm environments (159). Although electronic management of the farm environment strives to maintain constant conditions, fluctuations in the levels of the above listed environmental variables are still inevitable at various times throughout animals' lives (160). This is particularly evident in the case of environmental temperatures. Pigs are especially sensitive to heat stress, as they lack functional sweat glands and have a thick layer of adipose tissue which acts as insulation (51). Combining this vulnerability with the prolonged periods of increased environmental temperatures which sows experience in many pork producing regions (161), heat stress has the potential to act as a true chronic stress risk factor (51). In addition, with global warming on the rise, heat stress may become a problem of an increasingly chronic nature in places where until now it acted as an intermittent stressor. Moreover, heat stress in pregnant sows is linked with markedly reduced productivity and impaired reproductive performance [irregular expression of oestrous, reduced farrowing rates, increased abortion rates, and reduced litter size (88, 162)], greater inflammatory response at farrowing, and insulin resistance during lactation, all of which are indicative of a heightened chronic stress response (88).

### Individual Sow Factors

As outlined in Mechanisms underlying chronic stress, individual sows differ in personalities and coping styles and in how they adapt in response to stressors (63, 64). As different coping styles are associated with differential physiological responses to stress [e.g., higher expression of glucocorticoid receptors in proactive pigs, vs. higher oxytocin receptor expression in reactive pigs (163)], it is possible that each personality or coping style may act as a risk factor for the experience of chronic stress to a different extent (163). Indeed, personalities/coping styles exhibit temporal stability (164), and therefore those styles which are associated with a heightened stress response are an especially likely candidate to act as a chronic stress risk factor.

Little variation in body weight between sows within a group is another proposed risk factor for chronic stress, mediated by the potential to sustain high levels of aggression (52). Although the extent to which this is the case depends on the degree of body weight variation between sows, as well as other factors such as group size. Housing sows of unequal body weights together leads to reduced aggression levels at mixing (53), whereas sows of similar body weights could take longer to settle dominance conflicts, due to their evenly matched strength and fighting ability (165). Animals are able to assess the fighting ability (resource holding potential) of conspecifics, and based on the information gathered, decide whether to attack or withdraw (166). In general, smaller animals tend to avoid conflicts with larger individuals (52). It is therefore possible that a strategy of mixing sows of a range of sizes could also reduce long-term, sustained aggression levels. However, there is no research investigating the implications of sows housed with pen mates of equal or unequal body weights for cortisol concentrations.

The variation in parity of sows within a group must also be considered. Specifically, housing younger sows (parity 1 and 2) with older, multiparous sows (parity > 2) exacerbates aggression experienced by the former, generally subordinate and more vulnerable animals (167). Indeed, first parity sows housed with multiparous sows had lower farrowing rates compared to gilts housed with first parity sows only (167). This reflects the detrimental effect of housing sows of different parities on reproductive performance, mediated by the resulting increased aggression levels, and associated chronic stress.

Finally, the lack of familiarity between sows at mixing into groups may increase levels of aggression and therefore chronic stress (168). Previous studies demonstrate that improving familiarity among sows *via* pre-mixing (and thus sub-group establishment) prior to mixing reduces levels of aggression at mixing (168–170). For instance, no major disruption to social organisation and lower levels of aggression were observed when familiar sows were mixed together (168). This in turn could reduce levels of chronic stress (168).

## Prenatal Effects of Chronic Stress in Sows on Their Offspring

Various studies show the potential effects of prenatal stress on swine offspring, whether physiological or psychological (behaviour/personality). Specific physiological effects reported to date include altered development of the HPA axis (76), and associated decreased (86), or increased levels of basal circulating cortisol (171). Increased offspring hippocampal glucocorticoid receptors, decreased serum immunoglobulin G concentrations, and decreased lymphocyte proliferation (86) are also reported. Such effects can result in reduced immunity, higher susceptibility to disease, and greater mortality among prenatally stressed piglets (17). A potential effect of prenatal stress on offspring resilience was reported as a result of repeated nose sling restraint applied to sows during gestation, which more than doubled the mortality of neonatal piglets during the sucking period (17, 172). Similarly, Kanitz et al. (86) showed a higher frequency of disease and higher mortality during lactation in prenatally stressed piglets (born to sows restrained daily for 5 min, for a period of 5 weeks in late gestation), than in non-stressed piglets.

Prenatal stress can also have profound psychological effects on offspring, manifested as altered behaviour immediately after birth and throughout adult life (173), sometimes with sex-specific differences (174). Jarvis et al. (174) showed an effect of prenatal stress (a consequence of mixing stress imposed on pregnant sows) on female offspring, which displayed abnormal maternal behaviour later in life. This included restlessness and more responsiveness towards piglets that approached the sow's head, as well as a tendency to bite more at the piglets (174). Others showed that mothers that experienced pre-natal stress *in utero* spent more time lying ventrally following the birth of their first piglet, more time standing, and made more postural changes (175). These mothers also spent longer visually attending to their piglets compared to non-stressed mothers (175). This is suggestive of a pro-anxiety phenotype resulting from altered brain development during foetal life, as a consequence of mixing stress being imposed on the pregnant sow (175).

Other studies found effects of prenatal stress on behaviour regardless of sex. This included a heightened behavioural response to acute pain and injury such as tail docking (176), or decreased exploratory behaviour in a novel environment shown by prenatally stressed piglets born to sows repeatedly mixed throughout gestation to impose stress (177). Brajon et al. (177) also found decreased locomotion play and fighting play in prenatally stressed piglets, indicative of compromised welfare (178). In addition, the coping behaviour of prenatally stressed offspring is similar to the coping behaviour of humans with depression, suggesting that prenatally stressed offspring may also be at risk of developing depression-like symptoms (79).

Some studies investigating the effects of prenatal stress used models that artificially induce a stress response in sows, for example, through adrenocorticotrophic injections or cortisol administration (179, 180). In addition, many studies investigate only individual acute stressors (86, 175, 180), with a lack of focus on chronic stressors. While useful in providing knowledge on prenatal stress mechanisms, such studies lack on-farm applicability, and therefore their results cannot fully represent real-life scenarios.

Investigating the potential for chronic stress to result in prenatal stress is more applicable to real life situations that sows might experience. The risk factors for chronic stress discussed above are commonly found on-farm, often in combination, and are thus likely to be a realistic risk for prenatal stress in offspring. For instance, piglets of sows housed in barren environments had higher pre-weaning mortality (45, 181), and reduced neonatal survival (182), compared to piglets of sows housed with enrichment (deep straw bedding; manipulable wood materials and straw pellets). In the study of Quesnel et al. (183), piglets born to sows housed in non-enriched environments showed reduced maturity in terms of various physiological indicators at birth, compared to piglets born to sows from enriched housing, which is likely less stressful for the mother. Likewise, Tatemoto et al. (184) showed a beneficial effect of providing enrichment to sows during gestation on offspring behaviour outcomes. In that study, offspring born to sows from enriched environments showed less aggression and less nosing behaviour (184). In addition, female offspring specifically showed more exploratory behaviour and less fear during a novel object test (184).

As discussed above, lameness in sows is another potential chronic maternal stressor. Offspring born to lame sows have altered weight gain, aggressiveness, and also vocalisation levels during open field and novel object tests compared to piglets from non-lame sows (185, 186). Likewise, the offspring of restrictively fed sows on a low fibre diet showed more aggressive behaviour prior to weaning compared to the offspring of sows fed a high fibre diet (187). Heat stress also generates sufficient chronic stress in pregnant sows to lead to developmental damage to their offspring *in utero* (88); this includes altered offspring thermoregulatory ability (188), carcass composition (189), as well as sex-specific effects, such as reduced numbers of functional ovarian oocytes in female offspring (189), and reduced sperm number and quality in male offspring (190). Such findings confirm that exposing sows to a range of potential chronic stressors experienced during gestation does indeed cause a level of prenatal stress that has long-term, negative effects on offspring (183).

## Future Research

The aim of this review was to discuss the potential for several aspects of the sow physical and social environment to act as risk factors for chronic stress. Moreover, the review considered these risk factors in terms of their potential to cause prenatal stress in offspring. With increasing focus on improving animal welfare in recent years, the study of chronic stress and its consequences for the sow and her offspring is an area warranting urgent investigation. Chronic stress in gestation not only has immediate negative consequences for the sow [e.g., immunosuppression and associated morbidity (28, 29); and reduced reproductive performance (23, 24)], but also has long-term consequences for their offspring in terms of susceptibility to disease (191). Such negative effects not only reduce sow and piglet welfare (23, 191), but also threaten the sustainability of the pig industry. This is the case due to diminished sow reproductive performance (23), reduced piglet growth efficiency, and an increased need for the use of antimicrobials to treat disease in piglets, which has consequences for antibiotic resistance (192). Nevertheless, the extent to which a number of the factors discussed in the current review contribute to chronic stress in sows is still poorly understood, with even less research into their potential to contribute to prenatal stress and their postulated consequences for the offspring.

Due to numerous confounders (i.e., group size, quality and quantity of available space, or whether extra space is required by larger sows) which must be considered when investigating the effects of different space allowances on stress levels (93), an optimal space allowance, as well as the contribution of inadequate space allowances to chronic stress are not yet established for sows (94). Future study designs should take such confounders into consideration to ensure that results are meaningful in a broad context.

The absence of high fibre diet provision can exacerbate the chronic hunger effect and stress associated with restrictive feeding (40, 193, 194). Moreover, effective “off the floor” methods of roughage material delivery should be investigated. However, caution must be exercised when adopting “off the floor” methods such as straw racks, as such structures increased aggression associated with competition for access to the racks (195).

Enrichment materials are of interest and value to sows, and if not enough of them are provided, or they are difficult to access, they are usually monopolised by dominant animals. Further research should identify a method of enrichment delivery that ensures all animals have access, and which does not induce competition and stimulate aggression (145, 195). This will help to ensure that the positive effects of enrichment provision on sow stress levels are not counteracted by the negative effects of the aggression associated with competition for it.

There is potential in housing sows of unequal body weights together to reduce sustained aggression levels and the associated consequences related to chronic stress. Specifically, physiological measures such as cortisol concentration and immune status of individuals housed in groups with varying degrees of body weight variation should be measured to ascertain this possibility.

Given the benefits of rubber flooring to sow welfare overall (123, 146–149), indications of a reluctance to interact on such flooring, possibly attributable to slipperiness and fear of slipping/falling should be elucidated.

Studies investigating the link between stress resulting from the sow's fear of humans and the prenatal stress risks for her offspring are limited (64). Research in this area is needed to further highlight the importance of good quality stockmanship, and more effort must be committed to training of stock people to ensure their knowledge of this area (50).

## Conclusion

Chronic stress during gestation is not only detrimental to sow welfare and productivity, but also to their offspring, mediated by prenatal stress. The current review flagged a number of factors with potential to contribute to chronic stress in sows. There is an existing body of knowledge on methods to improve sow welfare during gestation, which could be used as a starting point to encourage pig industry stakeholders to adopt strategies to minimise levels of chronic stress experienced by sows. This could lead not only to positive associated effects for sow welfare and productivity, but also for the resilience and health of the offspring, and to increased societal acceptability of pig production. Nevertheless, several of the potential risk factors which can contribute to chronic stress still require additional research to determine the extent of their contribution, and also their potential to induce prenatal stress in offspring. Further investigation into these factors would also help to decide which sources of stress should be prioritised. Likewise, the impact of multiple concurrent chronic stressors also requires further investigation, as it is unlikely that any of the above reviewed chronic stress risk factors exist in isolation, with sows potentially experiencing multiple stressors at once. Although challenging, system based studies could be a potentially useful way of addressing this gap. Furthermore, such knowledge would help to determine whether to target sources of stress individually or in combination for an improved effect. Overall, novel research in the areas outlined in this review will be beneficial to sow and piglet welfare and productivity, with economically positive consequences for the pig industry.

## Author Contributions

ML: planned and organised the article, as well as drafted the manuscript. LB, JM, and KO'D: revised the manuscript. All authors contributed to the article and approved the submitted version.

## Funding

ML was funded by the Teagasc Walsh Scholarship Programme. The work was conducted as part of a Teagasc funded grant-in-aid project effects of chronic stress on sow welfare and performance, and on the resilience of their offspring (SowWeanWel) ref 0370.

## Conflict of Interest

The authors declare that the research was conducted in the absence of any commercial or financial relationships that could be construed as a potential conflict of interest.

## Publisher's Note

All claims expressed in this article are solely those of the authors and do not necessarily represent those of their affiliated organizations, or those of the publisher, the editors and the reviewers. Any product that may be evaluated in this article, or claim that may be made by its manufacturer, is not guaranteed or endorsed by the publisher.

## References

[1] Bench C Rioja-Lang F Hayne S Gonyou H. Group gestation sow housing with individual feeding—II: how space allowance, group size and composition, and flooring affect sow welfare. Livest Sci. (2013) 152:218–27. 10.1016/j.livsci.2012.12.020

[2] Horback K. 284 Prop 12 and Its Implications for Future on-Farm Animal Welfare in the United States. J Anim Sci. (2021) 99(Suppl. 1):8–9. 10.1093/jas/skab054.013

[3] Einarsson S Sjunnesson Y Hulten F Eliasson-Selling L Dalin AM Lundeheim N. A 25 years experience of group-housed sows-reproduction in animal welfare-friendly systems. Acta Vet Scand. (2014) 56:37. 10.1186/1751-0147-56-37 24910081 PMC4061533

[4] Andersen IL Boe KE Kristiansen AL. The Influence of different feeding arrangements and food type on competition at feeding in pregnant sows. Appl Anim Behav Sci. (1999) 65:91–104. 10.1016/S0168-1591(99)00058-1

[5] Anil L Anil SS Deen J Baidoo SK Wheaton JE. Evaluation of well-being, productivity, and longevity of pregnant sows housed in groups in pens with an electronic sow feeder or separately in gestation stalls. Am J Vet Res. (2005) 66:1630–8. 10.2460/ajvr.2005.66.1630 16261839

[6] Martinez-Miro S Tecles F Ramon M Escribano D Hernandez F Madrid J. Causes, consequences and biomarkers of stress in swine: an update. BMC Vet Res. (2016) 12:171 10.1186/s12917-016-0791-8 27543093 PMC4992232

[7] Bradshaw R Parrott R Goode J Lloyd D Rodway R Broom D. Behavioural and hormonal responses of pigs during transport: effect of mixing and duration of journey. Anim Sci. (1996) 62:547–54. 10.1017/S1357729800015095

[8] Huang Y Liu Z Liu W Yin C Ci L Zhao R. Salivary haptoglobin and chromogranin a as non-invasive markers during restraint stress in pigs. Res Vet Sci. (2017) 114:27–30. 10.1016/j.rvsc.2017.02.023 28284048

[9] Soler L Gutiérrez A Escribano D Fuentes M Cerón J. Response of salivary haptoglobin and serum amyloid a to social isolation and short road transport stress in pigs. Res Vet Sci. (2013) 95:298–302. 10.1016/j.rvsc.2013.03.007 23566790

[10] Otten W Puppe B Stabenow B Kanitz E Schön P Brüssow K. Agonistic interactions and physiological reactions of top-and bottom-ranking pigs confronted with a familiar and an unfamiliar group: preliminary results. Appl Anim Behav Sci. (1997) 55:79–90. 10.1016/S0168-1591(97)00036-1

[11] Merlot E Meunier-Salaün M-C Prunier A. Behavioural, endocrine and immune consequences of mixing in weaned piglets. Appl Anim Behav Sci. (2004) 85:247–57. 10.1016/j.applanim.2003.11.002

[12] Rushen J Pajor E. Offence and defence in fights between young pigs (Sus Scrofa). Aggress Behav. (1987) 13:329–46.

[13] Ruis MA de Groot J te Brake JH Ekkel ED van de Burgwal JA Erkens JH. Behavioural and physiological consequences of acute social defeat in growing gilts: effects of the social environment. Appl Anim Behav Sci. (2001) 70:201–25. 10.1016/S0168-1591(00)00150-7 11118662

[14] Emack J Kostaki A Walker CD Matthews SG. Chronic maternal stress affects growth, behaviour and hypothalamo-pituitary-adrenal function in juvenile offspring. Horm Behav. (2008) 54:514–20. 10.1016/j.yhbeh.2008.02.025 18674758

[15] Kapoor A Dunn E Kostaki A Andrews MH Matthews SG. Programming of hypothalamo-pituitary-adrenal function: prenatal stress and glucocorticoids. J Physiol-London. (2006) 572:31–44. 10.1113/jphysiol.2006.105254 16469780 PMC1779638

[16] Kranendonk G Mulder EJH Parvizi N Taverne MAM. Prenatal stress in pigs: experimental approaches and field observations. Exp Clin Endocrinol Diabetes. (2008) 116:413–22. 10.1055/s-2008-1065335 18484065

[17] Tuchscherer M Kanitz E Otten W Tuchscherer A. Effects of prenatal stress on cellular and humoral immune responses in neonatal pigs. Vet Immunol Immunopathol. (2002) 86:195–203. 10.1016/S0165-2427(02)00035-1 12007885

[18] Ladewig J. Chronic intermittent stress: a model for the study of long-term stressors. Biol Anim Stress. (2000). p. 159–69. 10.1079/9780851993591.0159

[19] Jarvis S D'Eath RB Robson SK Lawrence AB. The effect of confinement during lactation on the hypothalamic–pituitary–adrenal axis and behaviour of primiparous sows. Physiol Behav. (2006) 87:345–52. 10.1016/j.physbeh.2005.10.004 16332379

[20] van der Staay FJ Schuurman T Hulst M Smits M Prickaerts J Kenis G. Effects of chronic stress: a comparison between tethered and loose sows. Physiol Behav. (2010) 100:154–64. 10.1016/j.physbeh.2010.02.020 20193701

[21] Salak-Johnson JL. Social status and housing factors affect reproductive performance of pregnant sows in groups. Mol Reprod Dev. (2017) 84:905–13. 10.1002/mrd.22846 28763574

[22] Braastad BO. Effects of prenatal stress on behaviour of offspring of laboratory and farmed mammals. Appl Anim Behav Sci. (1998) 61:159–80. 10.1016/S0168-1591(98)00188-9

[23] Spoolder HAM Geudeke MJ Van der Peet-Schwering CMC Soede NM. Group housing of sows in early pregnancy: a review of success and risk factors. Livest Sci. (2009) 125:1–14. 10.1016/j.livsci.2009.03.009

[24] Einarsson S Brandt Y Lundeheim N Madej A. Stress and its influence on reproduction in pigs: a review. Acta Vet Scand. (2008) 50:48. 10.1186/1751-0147-50-48 19077201 PMC2630310

[25] Kick AR Tompkins MB Almond GW. Stress and immunity in the pig. Anim Sci Rev. (2011) 212:51–65. 10.1079/PAVSNNR20116018

[26] Moberg GP Mench JA. The Biology of Animal Stress: Basic Principles and Implications for Animal Welfare. CABI (2000). 10.1079/9780851993591.0001

[27] Ruis MA te Brake JH Engel B Buist WG Blokhuis HJ Koolhaas JM. Adaptation to social isolation: acute and long-term stress responses of growing gilts with different coping characteristics. Physiol Behav. (2001) 73:541–51. 10.1016/S0031-9384(01)00548-0 11495658

[28] Olsson IAS de Jonge FH Schuurman T Helmond FA. Poor rearing conditions and social stress in pigs: repeated social challenge and the effect on behavioural and physiological responses to stressors. Behav Processes. (1999) 46:201–15. 10.1016/S0376-6357(99)00036-4 24896444

[29] Salak-Johnson JL McGlone JJ. Making sense of apparently conflicting data: stress and immunity in swine and cattle. J Anim Sci. (2007) 85:E81–8. 10.2527/jas.2006-538 17085721

[30] Carroll GA Boyle LA Hanlon A Palmer MA Collins L Griffin K. Identifying physiological measures of lifetime welfare status in pigs: exploring the usefulness of haptoglobin, C- reactive protein and hair cortisol sampled at the time of slaughter. Ir Vet J. (2018) 71:8. 10.1186/s13620-018-0118-0 29507716 PMC5833096

[31] Herman J McKlveen J Ghosal S Kopp B Wulsin A Makinson R. Regulation of the hypothalamic-pituitary-adrenocortical stress response. Compr Physiol. (2016) 6:603–21. 10.1002/cphy.c150015 27065163 PMC4867107

[32] Davenport MD Tiefenbacher S Lutz CK Novak MA Meyer JS. Analysis of endogenous cortisol concentrations in the hair of rhesus macaques. Gen Comp Endocrinol. (2006) 147:255–61. 10.1016/j.ygcen.2006.01.005 16483573

[33] Heimburge S Kanitz E Otten W. The use of hair cortisol for the assessment of stress in animals. Gen Comp Endocrinol. (2019) 270:10–7. 10.1016/j.ygcen.2018.09.016 30287191

[34] Meyer JS Novak MA. Minireview: hair cortisol: a novel biomarker of hypothalamic-pituitary-adrenocortical activity. Endocrinology. (2012) 153:4120–7. 10.1210/en.2012-1226 22778226 PMC3423616

[35] Nemeth M Pschernig E Wallner B Millesi E. Non-invasive cortisol measurements as indicators of physiological stress responses in guinea pigs. PeerJ. (2016) 4:e1590. 10.7717/peerj.1590 26839750 PMC4734438

[36] Barnett J Hemsworth P Cronin G Newman E McCallum T Chilton D. Effects of pen size, partial stalls and method of feeding on welfare-related behavioural and physiological responses of group-housed pigs. Appl Anim Behav Sci. (1992) 34:207–20. 10.1016/S0168-1591(05)80116-9

[37] Hemsworth P Barnett J Hansen C Winfield C. Effects of social environment on welfare status and sexual behaviour of female pigs. II effects of space allowance. Appl Anim Behav Sci. (1986) 16:259–67. 10.1016/0168-1591(86)90118-8

[38] Grimberg-Henrici C Büttner K Ladewig R Burfeind O Krieter J. Cortisol levels and health indicators of sows and their piglets living in a group-housing and a single-housing system. Livest Sci. (2018) 216:51–60. 10.1016/j.livsci.2018.07.006

[39] Amdi C Giblin L Hennessy A Ryan T Stanton C Stickland N. Feed allowance and maternal backfat levels during gestation influence maternal cortisol levels, milk fat composition and offspring growth. J Nutr Sci. (2013) 2:e1. 10.1017/jns.2012.20 25191557 PMC4153285

[40] Meunier-Salaun MC Edwards SA Robert S. Effect of dietary fibre on the behaviour and health of the restricted fed sow. Anim Feed Sci Technol. (2001) 90:53–69. 10.1016/S0377-8401(01)00196-1

[41] Contreras-Aguilar MD Escribano D Martinez-Miro S Lopez-Arjona M Rubio CP Martinez-Subiela S. Application of a score for evaluation of pain, distress and discomfort in pigs with lameness and prolapses: correlation with saliva biomarkers and severity of the disease. Res Vet Sci. (2019) 126:155–63. 10.1016/j.rvsc.2019.08.004 31494378

[42] Escribano D Horvatić A Contreras-Aguilar MD Guillemin N Cerón JJ Tecles F. Changes in saliva proteins in two conditions of compromised welfare in pigs: an experimental induced stress by nose snaring and lameness. Res Vet Sci. (2019) 125:227–34. 10.1016/j.rvsc.2019.06.008 31284225

[43] Barnett JL Cronin GM McCallum TH Newman EA Hennessy DP. Effects of grouping unfamiliar adult pigs after dark, after treatment with amperozide and by using pens with stalls, on aggression, skin lesions and plasma cortisol concentrations. Appl Anim Behav Sci. (1996) 50:121–33. 10.1016/0168-1591(96)01084-2

[44] Bahnsen I Riddersholm K de Knegt L Bruun T Amdi C. The effect of different feeding systems on salivary cortisol levels during gestation in sows on herd level. Animals. (2021) 11:1074. 10.3390/ani11041074 33918923 PMC8070664

[45] Merlot E Calvar C Prunier A. Influence of the housing environment during sow gestation on maternal health, and offspring immunity and survival. Anim Prod Sci. (2017) 57:1751–8. 10.1071/AN15480

[46] Boyle L Moya SL. Effect of covering slatted floors with mats on the behaviour and welfare of loose housed sows at mixing. In: Proceedings of the 37th International Congress of the International Society for Applied Ethology. Abano Terme: ISAE (2003).

[47] Milne F Dudgeon M Valli V. Springing sole syndrome. Mod Vet Pract. (1974) 55:48–51. 4811094

[48] Walker N Beattie V. Welfare of Sows in Loose Housed Systems. 67th Annual Report of the Agricultural Research Institute of Northern Ireland. Hillsborough, UK; Agri-Food and Biosciences Institute (1994).

[49] Boivin X Lensink J Tallet C Veissier I. Stockmanship and farm animal welfare. Anim Welf. (2003) 12:479–92.

[50] Manteca X Jones B. Welfare improvement strategies. Improving Farm Anim Welf. (2013). p. 175–200. 10.3920/978-90-8686-770-7_8

[51] Ross J Hale B Gabler N Rhoads R Keating A Baumgard L. Physiological consequences of heat stress in pigs. Anim Prod Sci. (2015) 55:1381–90. 10.1071/AN15267

[52] Arey DS Edwards SA. Factors influencing aggression between sows after mixing and the consequences for welfare and production. Livest Prod. Sci. (1998) 56:61–70. 10.1016/S0301-6226(98)00144-4

[53] Gonyou H Baker A Rhode K Petrie C. Aggression and production in growing pigs in groups of uniform or varying weights. J Anim Sci. (1983) 57(Suppl. 1):137.

[54] Golbidi S Frisbee JC Laher I. Chronic stress impacts the cardiovascular system: animal models and clinical outcomes. Am J Physiol Heart Circ Physiol. (2015) 308:H1476–98. 10.1152/ajpheart.00859.2014 25888514

[55] Selye H. Stress and the general adaptation syndrome. Br Med J. (1950) 1:1383. 10.1136/bmj.1.4667.1383 15426759 PMC2038162

[56] Moberg GP. Influence of stress on reproduction: measure of well-being. In: Animal stress. New York, NY: Springer (1985). p. 245–67. 10.1007/978-1-4614-7544-6_14

[57] Morgan L Meyer J Novak S Younis A Ahmad W Raz T. Shortening sow restraint period during lactation improves production and decreases hair cortisol concentrations in sows and their piglets. Animal. (2021) 15:100082. 10.1016/j.animal.2020.100082 33509702

[58] Won E Kim Y. Stress, the autonomic nervous system, and the immune-kynurenine pathway in the etiology of depression. Curr Neuropharmacol. (2016) 14:665–73. 10.2174/1570159X14666151208113006 27640517 PMC5050399

[59] Otovic P Hutchinson E. Limits to using Hpa axis activity as an indication of animal welfare. ALTEX. (2015) 32:41–50. 10.14573/altex.1406161 25418851

[60] Lagoda M O'Driscoll K Marchewka J Foister S Turner S Boyle L. Associations between skin lesion counts, hair cortisol concentrations and reproductive performance in group housed sows. Livest Sci. (2021) 246:104463. 10.1016/j.livsci.2021.104463

[61] Dickens M Romero L. A consensus endocrine profile for chronically stressed wild animals does not exist. Gen Comp Endocrinol. (2013) 191:177–89. 10.1016/j.ygcen.2013.06.014 23816765

[62] Cockrem JF. individual variation in glucocorticoid stress responses in animals. Gen Comp Endocrinol. (2013) 181:45–58. 10.1016/j.ygcen.2012.11.025 23298571

[63] Koolhaas J De Boer S Coppens C Buwalda B. Neuroendocrinology of coping styles: towards understanding the biology of individual variation. Front Neuroendocrinol. (2010) 31:307–21. 10.1016/j.yfrne.2010.04.001 20382177

[64] Rooney B Schmitt O Courty A Lawlor P O'Driscoll K. Like mother like child: do fearful sows have fearful piglets? Animals. (2021) 11:1232. 10.3390/ani11051232 33923259 PMC8146394

[65] Bhatnagar S Dallman M. Neuroanatomical basis for facilitation of hypothalamic-pituitary-adrenal responses to a novel stressor after chronic stress. Neuroscience. (1998) 84:1025–39. 10.1016/S0306-4522(97)00577-0 9578393

[66] Herman J. Neural control of chronic stress adaptation. Front Behav Neurosci. (2013) 7:61. 10.3389/fnbeh.2013.00061 23964212 PMC3737713

[67] Marti O Gavaldà A Gómez F Armario A. Direct evidence for chronic stress-induced facilitation of the adrenocorticotropin response to a novel acute stressor. Neuroendocrinology. (1994) 60:1–7. 10.1159/000126713 8090276

[68] Rich EL Romero LM. Exposure to chronic stress downregulates corticosterone responses to acute stressors. Am J Physiol Regul Integr Comp Physiol. (2005) 288:R1628–36. 10.1152/ajpregu.00484.2004 15886358

[69] Welberg LA Thrivikraman K Plotsky PM. Chronic maternal stress inhibits the capacity to up-regulate placental 11β-hydroxysteroid dehydrogenase type 2 activity. J Endocrinol. (2005) 186:R7–12. 10.1677/joe.1.06374 16135661

[70] Ijichi C Collins L Elwood R. Evidence for the role of personality in stereotypy predisposition. Anim Behav. (2013) 85:1145–51. 10.1016/j.anbehav.2013.03.033

[71] Lebelt D Zanella A Unshelm J. Physiological correlates associated with cribbing behaviour in horses: changes in thermal threshold, heart rate, plasma B-endorphin and serotonin. Equine Vet J. (1998) 30:21–7. 10.1111/j.2042-3306.1998.tb05140.x 10484999

[72] Sarrafchi A Blokhuis H. Equine stereotypic behaviors: causation, occurrence, and prevention. J Vet Behav. (2013) 8:386–94. 10.1016/j.jveb.2013.04.068

[73] McBride S Cuddeford D. The putative welfare-reducing effects of preventing equine stereotypic behavior. Anim Welfare. (2001) 10:173–89.

[74] Pomerantz O Paukner A Terkel J. Some stereotypic behaviors in rhesus macaques (Macaca Mulatta) are correlated with both perseveration and the ability to cope with acute stressors. Behav Brain Res. (2012) 230:274–80. 10.1016/j.bbr.2012.02.019 22366267 PMC3635133

[75] Mason GJ Latham NR. Can't stop, won't stop: is stereotypy a reliable animal welfare indicator?. Anim Welfare. (2004) 13:S57–69.

[76] Otten W Kanitz E Tuchscherer M Nurnberg G. Effects of prenatal restraint stress on hypothalamic-pituitary-adrenocortical and sympatho-adrenomedullary axis in neonatal pigs. Anim Sci. (2001) 73:279–87. 10.1017/S1357729800058252

[77] Grigsby P. Animal models to study placental development and function throughout normal and dysfunctional human pregnancy. Semin Reprod Med. (2016) 34:11–6. 10.1055/s-0035-1570031 26752715 PMC4799492

[78] Furukawa S Kuroda Y Sugiyama A. A comparison of the histological structure of the placenta in experimental animals. J Toxicol Pathol. (2014) 27:11–8. 10.1293/tox.2013-0060 24791062 PMC4000068

[79] Weinstock M. Does prenatal stress impair coping and regulation of hypothalamic-pituitary-adrenal axis? Neurosci Biobehav Rev. (1997) 21:1–10. 10.1016/S0149-7634(96)00014-0 8994205

[80] Salomon S Bejar C Schorer-Apelbaum D Weinstock M. Corticosterone mediates some but not other behavioural changes induced by prenatal stress in rats. J Neuroendocrinol. (2011) 23:118–28. 10.1111/j.1365-2826.2010.02097.x 21108672

[81] Jašarević E Howard C Morrison K Misic A Weinkopff T Scott P. The maternal vaginal microbiome partially mediates the effects of prenatal stress on offspring gut and hypothalamus. Nat Neurosci. (2018) 21:1061–71. 10.1038/s41593-018-0182-5 29988069

[82] Brunton P. Resetting the dynamic range of hypothalamic-pituitary-adrenal axis stress responses through pregnancy. J Neuroendocrinol. (2010) 22:1198–213. 10.1111/j.1365-2826.2010.02067.x 20819122

[83] Merlot E Couret D Otten W. Prenatal stress, fetal imprinting and immunity. Brain Behav Immun. (2008) 22:42–51. 10.1016/j.bbi.2007.05.007 17716859

[84] Couret D Jamin A Kuntz-Simon G Prunier A Merlot E. Maternal stress during late gestation has moderate but long-lasting effects on the immune system of the piglets. Vet Immunol Immunopathol. (2009) 131:17–24. 10.1016/j.vetimm.2009.03.003 19362376

[85] Couret D Prunier A Mounier AM Thomas F Oswald IP Merlot E. Comparative effects of a prenatal stress occurring during early or late gestation on pig immune response. Physiol Behav. (2009) 98:498–504. 10.1016/j.physbeh.2009.08.003 19686768

[86] Kanitz E Otten W Tuchscherer M Manteuffel G. Effects of prenatal stress on corticosteroid receptors and monoamine concentrations in limbic areas of suckling piglets (Sus Scrofa) at different ages. J Vet Med A Physiol Pathol Clin Med. (2003) 50:132–9. 10.1046/j.1439-0442.2003.00513.x 12757550

[87] Omtvedt I Nelson R Edwards R Stephens D Turman E. Influence of heat stress during early, mid and late pregnancy of gilts. J Anim Sci. (1971) 32:312–7. 10.2527/jas1971.322312x 5543028

[88] Lucy M Safranski T. Heat stress in pregnant sows: thermal responses and subsequent performance of sows and their offspring. Mol Reprod Dev. (2017) 84:946–56. 10.1002/mrd.22844 28696547

[89] Kongsted AG. Stress and fear as possible mediators of reproduction problems in group housed sows: a review. Acta Agric Scand Section A Anim Sci. (2004) 54:58–66. 10.1080/09064700410032031

[90] Brake JHT Bressers HP. Applications in service management and oestrus detection. In: Electronic Identification in Pig Production, Vol. 10. (1990). p. 63–7.

[91] Lagoda M Boyle L Marchewka J Calderón Díaz J. Mixing aggression intensity is associated with age at first service and floor type during gestation, with implications for sow reproductive performance. Animal. (2021) 15:100158. 10.1016/j.animal.2020.100158 33573987

[92] Weng RC Edwards SA English PR. Behaviour, social interactions and lesion scores of group-housed sows in relation to floor space allowance. Appl Anim Behav Sci. (1998) 59:307–16. 10.1016/S0168-1591(97)00143-3

[93] Barnett JL Hemsworth PH Cronin GM Jongman EC Hutson GD. A review of the welfare issues for sows and piglets in relation to housing. Aust J Agri Res. (2001) 52:1–28. 10.1071/AR00057

[94] Hemsworth PH Rice M Nash J Giri K Butler KL Tilbrook AJ. Effects of group size and floor space allowance on grouped sows: aggression, stress, skin injuries, and reproductive performance. J Anim Sci. (2013) 91:4953–64. 10.2527/jas.2012-5807 23893983

[95] Sundh H Finne-Fridell F Ellis T Taranger GL Niklasson L Pettersen EF. Reduced water quality associated with higher stocking density disturbs the intestinal barrier functions of atlantic salmon (Salmo Salar L.). Aquaculture. (2019) 512:734356. 10.1016/j.aquaculture.2019.734356

[96] Selvam R Saravanakumar M Suresh S Sureshbabu G Sasikumar M Prashanth D. Effect of vitamin E supplementation and high stocking density on the performance and stress parameters of broilers. Braz J Poult Sci. (2017) 19:587–94. 10.1590/1806-9061-2016-0417

[97] Schubach KM Cooke RF Brandao AP Lippolis KD Silva LGT Marques RS. Impacts of stocking density on development and puberty attainment of replacement beef heifers. Animal. (2017) 11:2260–7. 10.1017/S1751731117001070 28521848

[98] Mack LA Lay DC Eicher SD Johnson AK Richert BT Pajor EA. Group space allowance has little effect on sow health, productivity, or welfare in a free-access stall system. J Anim Sci. (2014) 92:2554–67. 10.2527/jas.2013-7352 24668955

[99] Kelley KW Mcglone JJ Gaskins CT. Porcine aggression - measurement and effects of crowding and fasting. J Anim Sci. (1980) 50:336–41. 10.2527/jas1980.502336x 7192279

[100] Velarde A. Agonistic Behaviour. On Farm Monitoring of Pig Welfare. Wageningen: Wageningen Academic Publishers (2007).

[101] Greenwood EC Plush KJ van Wettere WHEJ Hughes PE. Group and individual sow behavior is altered in early gestation by space allowance in the days immediately following grouping. J Anim Sci. (2016) 94:385–93. 10.2527/jas.2015-9427 26812343

[102] Chapinal N Ruiz-De-La-Torre JL Cerisuelo A Baucells MD Gasa J Manteca X. Feeder use patterns in group-housed pregnant sows fed with an unprotected electronic sow feeder (Fitmix). J Appl Anim Welf Sci. (2008) 11:319–36. 10.1080/10888700802329939 18821401

[103] Simmins PH. Reproductive-performance of sows entering stable and dynamic groups after mating. Anim Prod. (1993) 57:293–8. 10.1017/S0003356100006917

[104] Anil L Anil SS Deen J Baidoo SK Walker RD. Effect of group size and structure on the welfare and performance of pregnant sows in pens with electronic sow feeders. Can J Vet Res. (2006) 70:128–36. 16639945 PMC1410729

[105] Couret D Otten W Puppe B Prunier A Merlot E. Behavioural, endocrine and immune responses to repeated social stress in pregnant gilts. Animal. (2009) 3:118–27. 10.1017/S1751731108003236 22444178

[106] Misra S Bokkers EA Upton J Quinn AJ O'Driscoll K. Effect of environmental enrichment and group size on the water use and waste in grower-finisher pigs. Sci Rep. (2021) 11:1–10. 10.1038/s41598-021-95880-0 34385550 PMC8361099

[107] Taylor IA Barnett JL Cronin GM. Optimum group size for pigs. In: Bottcher RW Hoff SJ editors. Proc. 5th. Int. Symp. Am. Soc. Agri. Eng. Livest. Environ.. MI (1997). p. 965–71.

[108] Peltoniemi OAT Tast A Love RJ. Factors effecting reproduction in the pig: seasonal effects and restricted feeding of the pregnant gilt and sow. Anim Reprod Sci. (2000) 60:173–84. 10.1016/S0378-4320(00)00092-0 10844193

[109] Verdon M Hansen CF Rault JL Jongman E Hansen LU Plush K. Effects of group housing on sow welfare: a review. J Anim Sci. (2015) 93:1999–2017. 10.2527/jas.2014-8742 26020296

[110] Schenkel AC Bernardi ML Bortolozzo FP Wentz I. Body reserve mobilization during lactation in first parity sows and its effect on second litter size. Livest Sci. (2010) 132:165–72. 10.1016/j.livsci.2010.06.002

[111] Zak LJ Cosgrove JR Aherne FX Foxcroft GR. Pattern of feed intake and associated metabolic and endocrine changes differentially affect postweaning fertility in primiparous lactating sows. J Anim Sci. (1997) 75:208–16. 10.2527/1997.751208x 9027568

[112] D'Eath RB Jarvis S Baxter EM Houdijk J. Mitigating hunger in pregnant sows. In: Advances in Pig Welfare. Woodhead Publishing (2018). p. 199–234. 10.1016/B978-0-08-101012-9.00007-1

[113] Stewart C Boyle L McCann M O'Connell N. The effect of feeding a high fibre diet on the welfare of sows housed in large dynamic groups. Anim Welfare. (2010) 19:349–57. Available online at: https://www.ingentaconnect.com/content/ufaw/aw/2010/00000019/00000003/art00016

[114] Sapkota A Marchant-Forde J Richert B Lay D Jr. Including dietary fiber and resistant starch to increase satiety and reduce aggression in gestating sows. J Anim Sci. (2016) 94:2117–27. 10.2527/jas.2015-0013 27285708

[115] Lawrence A Appleby M Macleod H. Measuring Hunger in the pig using operant conditioning: the effect of food restriction. Anim Sci. (1988) 47:131–7. 10.1017/S0003356100037132

[116] Brouns F Edwards SA English PR. The effect of dietary inclusion of sugar-beet pulp on the feeding behaviour of dry sows. Anim Sci. (1997) 65:129–33. 10.1017/S1357729800016386

[117] Bench CJ Rioja-Lang FC Hayne SM Gonyou HW. Group gestation housing with individual feeding-I: how feeding regime, resource allocation, and genetic factors affect sow welfare. Livest Sci. (2013) 152:208–17. 10.1016/j.livsci.2012.12.021

[118] Broom D. Stereotypies as Animal Welfare Indicators. Indicators Relevant to Farm Animal Welfare. Dordrecht: Springer (1983).

[119] De Leeuw JA Ekkel ED. Effects of feeding level and the presence of a foraging substrate on the behaviour and stress physiological response of individually housed gilts. Appl Anim Behav Sci. (2004) 86:15–25. 10.1016/j.applanim.2003.12.004

[120] Toscano MJ Lay DC Craig BA Pajor EA. Assessing the adaptation of swine to fifty-seven hours of feed deprivation in terms of behavioral and physiological responses. J Anim Sci. (2007) 85:441–51. 10.2527/jas.2006-316 17235029

[121] de Jong IC van Voorst S Ehlhardt DA Blokhuis HJ. Effects of restricted feeding on physiological stress parameters in growing broiler breeders. Br Poult Sci. (2002) 43:157–68. 10.1080/00071660120121355 12047078

[122] Anil SS Anil L Deen J. Effect of lameness on sow longevity. J Am Vet Med Assoc. (2009) 235:734–8. 10.2460/javma.235.6.734 19751172

[123] Calderon Diaz JA Fahey AG KilBride AL Green LE Boyle LA. Longitudinal study of the effect of rubber slat mats on locomotory ability, body, limb and claw lesions, and dirtiness of group housed sows. J Anim Sci. (2013) 91:3940–54. 10.2527/jas.2012-5913 23881683

[124] Dewey CE Friendship RM Wilson MR. Clinical and postmortem examination of sows culled for lameness. Can Vet J. (1993) 34:555–6. 17424287 PMC1686573

[125] Pell AN. Manure and microbes: public and animal health problem? J Dairy Sci. (1997) 80:2673–81. 10.3168/jds.S0022-0302(97)76227-1 9361239 PMC7130904

[126] Conte S Bergeron R Gonyou H Brown J Rioja-Lang FC Connor ML. Use of an analgesic to identify pain-related indicators of lameness in sows. Livest Sci. (2015) 180:203–8. 10.1016/j.livsci.2015.08.009

[127] Mustonen K Ala-Kurikka E Orro T Peltoniemi O Raekallio M Vainio O. Oral ketoprofen is effective in the treatment of non-infectious lameness in sows. Vet J. (2011) 190:55–9. 10.1016/j.tvjl.2010.09.017 21035362

[128] Iida R Piñeiro C Koketsu Y. Removal of sows in Spanish breeding herds due to lameness: incidence, related factors and reproductive performance of removed sows. Prev Vet Med. (2020) 179:105002. 10.1016/j.prevetmed.2020.105002 32388036

[129] Creed F. Psychological disorders in rheumatoid arthritis: a growing consensus? Ann Rheum Dis. (1990) 49:808–12. 10.1136/ard.49.10.808 2241274 PMC1004240

[130] Barnett J Bottcher R Hoff S. Modifying the design of group pens with individual feeding places affects the welfare of pigs. In: Bottcher RW Hoff SJ editors. Fifth International Livestock Environment Symposium. St. Joseph, MI: ILES X (1997).

[131] Luescher U Friendship R McKeown D. Evaluation of methods to reduce fighting among regrouped gilts. Can J Anim Sci. (1990) 70:363–70. 10.4141/cjas90-048

[132] Petherick JC Blackshaw JK. A review of the factors influencing the aggressive and agonistic behaviour of the domestic pig. Aust J Exp Agric. (1987) 27:605–11. 10.1071/EA9870605

[133] Edwards SA Mauchline S Stewart AH. Designing pens to minimize aggression when sows are mixed. Farm Build Progress (1993) 113:20–3.

[134] Spoolder H Corning S Edwards S. Welfare of finishing pigs after mixing in kennelled or unkennelled accommodation. Anim Sci. (1998). p. 118. 10.1017/S0308229600033316

[135] Spoolder H Lawrence A Edwards S Simmins P Armsby A. The effects of food level on the spatial organization of dynamic groups of sows. In; Proceedings of the Annual Meeting of the International Society for Applied Ethology, Clermont-Ferrand, France (1998).

[136] Bornett H Morgan C Lawrence A Mann J. The effect of group housing on feeding patterns and social behaviour of previously individually housed growing pigs. Appl Anim Behav Sci. (2000) 70:127–41. 10.1016/S0168-1591(00)00146-5 11080556

[137] Schubbert A Spoolder HA Pedersen LJ. Review on group housing and mixing of sows. EURCAW-Pigs. (2020). Available online at: https://edepot.wur.nl/537021

[138] Verdon M Rault J-L. Aggression in Group Housed Sows and Fattening Pigs. Spinka M editor. Advances in Pig Welfare. United Kingdom; Elsevier (2017).

[139] Brajon S Ahloy-Dallaire J Devillers N Guay F. Social status and previous experience in the group as predictors of welfare of sows housed in large semi-static groups. PLoS ONE. (2021) 16:e0244704. 10.1371/journal.pone.0244704 34101733 PMC8186791

[140] Gieling ET Nordquist RE van der Staay FJ. Assessing learning and memory in pigs. Anim Cogn. (2011) 14:151–73. 10.1007/s10071-010-0364-3 21203792 PMC3040303

[141] Studnitz M Jensen MB Pedersen LJ. Why do pigs root and in what will they root?: a review on the exploratory behaviour of pigs in relation to environmental enrichment. Appl Anim Behav Sci. (2007) 107:183–97. 10.1016/j.applanim.2006.11.0138785959

[142] Oostindjer M van den Brand H Kemp B Bolhuis JE. Effects of environmental enrichment and loose housing of lactating sows on piglet behaviour before and after weaning. Appl Anim Behav Sci. (2011) 134:31–41. 10.1016/j.applanim.2011.06.011 20622185

[143] Beattie V Sneddon I Walker N Weatherup R. Environmental enrichment of intensive pig housing using spent mushroom compost. Anim Sci. (2001) 72:35–42. 10.1017/S1357729800055533

[144] Vanheukelom V Driessen B Maenhout D Geers R. Peat as Environmental enrichment for piglets: the effect on behaviour, skin lesions and production results. Appl Anim Behav Sci. (2011) 134:42–7. 10.1016/j.applanim.2011.06.010

[145] Horback KM Pierdon MK Parsons TD. Behavioral preference for different enrichment objects in a commercial sow herd. Appl Anim Behav Sci. (2016) 184:7–15. 10.1016/j.applanim.2016.09.002

[146] Boyle LA Regan D Leonard FC Lynch PB Brophy P. The effect of mats on the welfare of sows and piglets in the farrowing house. Anim Welfare. (2000) 9:39–48. Available online at: https://www.ingentaconnect.com/content/ufaw/aw/2000/00000009/00000001/art00004

[147] Calderon Diaz JA Boyle LA. Effect of rubber slat mats on the behaviour and welfare of group housed pregnant sows. Appl Anim Behav Sci. (2014) 151:13–23. 10.1016/j.applanim.2013.11.016

[148] Elmore MRP Garner JP Johnson AK Richert BT Pajor EA A. Flooring comparison: the impact of rubber mats on the health, behavior, and welfare of group-housed sows at breeding. Appl Anim Behav Sci. (2010) 123:7–15. 10.1016/j.applanim.2009.11.012

[149] Tuyttens FAM Wouters F Struelens E Sonck B Duchateau L. Synthetic lying mats may improve lying comfort of gestating sows. Appl Anim Behav Sci. (2008) 114:76–85. 10.1016/j.applanim.2008.01.015

[150] Ostovic M Rafaj RB Mencik S Kabalin AE Grahovac J Matkovic K. The effect of rubber slat mats on cortisol concentrations in stall-housed gilts. Vet Arh. (2017) 87:185–96. Available online at: http://www.vef.hr/vetarhiv

[151] Barnett JL Cronin GM Mccallum TH Newman EA. Effects of pen size shape and design on aggression when grouping unfamiliar adult-pigs. Appl Anim Behav Sci. (1993) 36:111–22. 10.1016/0168-1591(93)90003-8

[152] Hemsworth P Coleman G. Human-Livestock Interactions-the stockperson and the productivity and welfare of intensively farmed animals. J S Afr Vet Assoc. (1998) 69:92. 10.4102/jsava.v69i3.832

[153] Hayes ME Hemsworth LM Morrison RS Butler KL Rice M Rault JL. Effects of positive human contact during gestation on the behaviour, physiology and reproductive performance of sows. Animals. (2021) 11:214. 10.3390/ani11010214 33467148 PMC7830568

[154] Dokmanovic M Velarde A Tomovic V Glamoclija N Markovic R Janjic J. The effects of lairage time and handling procedure prior to slaughter on stress and meat quality parameters in pigs. Meat Sci. (2014) 98:220–6. 10.1016/j.meatsci.2014.06.003 24971810

[155] Hemsworth PH Boivin X. Human Contact. Anim Welfare. (2011) 2:246–62. 10.1079/9781845936594.0246

[156] Jones RB. Fear and distress. In: Appleby MC Hughes BO editors. Animal Welfare. Wallingford: CAB International (1997). p. 75–87.

[157] Rushen J Taylor AA de Passillé AM. Domestic animals' fear of humans and its effect on their welfare. Appl Anim Behav Sci. (1999) 65:285–303. 10.1016/S0168-1591(99)00089-1

[158] Pedersen L Damm B Kongsted A. The influence of adverse or gentle handling procedures on sexual behaviour in fearful and confident sows. Appl Anim Behav Sci. (2003) 83:277–90. 10.1016/S0168-1591(03)00140-0

[159] Crook B Robertson J Glass ST Botheroyd E Lacey J Topping M. Airborne dust, ammonia, microorganisms, and antigens in pig confinement houses and the respiratory health of exposed farm workers. Am Ind Hyg Assoc J. (1991) 52:271–9. 10.1080/15298669191364721 1951065

[160] Fournel S Rousseau AN Laberge B. Rethinking environment control strategy of confined animal housing systems through precision livestock farming. Biosys Eng. (2017) 155:96–123. 10.1016/j.biosystemseng.2016.12.005

[161] Johnson J Baumgard L. Physiology symposium: postnatal consequences of in utero heat stress in pigs. J Anim Sci. (2019) 97:962–71. 10.1093/jas/sky472 30534960 PMC6358235

[162] Nardone A Ronchi B Lacetera N Bernabucci U. Climatic effects on productive traits in livestock. Vet Res Commun. (2006) 30:75. 10.1007/s11259-006-0016-x

[163] Kanitz E Tuchscherer M Otten W Tuchscherer A Zebunke M Puppe B. Coping style of pigs is associated with different behavioral, neurobiological and immune responses to stressful challenges. Front Behav Neurosci. (2019) 13:173. 10.3389/fnbeh.2019.00173 31417378 PMC6686684

[164] Horback K Parsons T. Temporal stability of personality traits in group-housed gestating sows. Animal. (2016) 10:1351–9. 10.1017/S1751731116000215 26915682

[165] Camerlink I Turner SP Farish M Arnott G. Aggressiveness as a component of fighting ability in pigs using a game-theoretical framework. Anim Behav. (2015) 108:183–91. 10.1016/j.anbehav.2015.07.032

[166] Arnott G Elwood RW. Assessment of fighting ability in animal contests. Anim Behav. (2009) 77:991–1004. 10.1016/j.anbehav.2009.02.010

[167] Li Y Wang L Johnston L. Sorting by parity to reduce aggression toward first-parity sows in group-gestation housing systems. J Anim Sci. (2012) 90:4514–22. 10.2527/jas.2011-4869 22859765

[168] Greenwood EC Plush KJ van Wettere WH Hughes PE. Hierarchy formation in newly mixed, group housed sows and management strategies aimed at reducing its impact. Appl Anim Behav Sci. (2014) 160:1–11. 10.1016/j.applanim.2014.09.011

[169] Durrell J Beattie V Sneddon I Kilpatrick D. Pre-Mixing as a Technique for Facilitating Subgroup Formation and Reducing Sow Aggression in Large Dynamic Groups. Appl Anim Behav Sci. (2003) 84:89–99. 10.1016/j.applanim.2003.06.001

[170] Pierdon MK Parsons TD. Effect of familiarity and mixing method on gestating sow welfare and productivity in large dynamic groups. J Anim Sci. (2018) 96:5024–34. 10.1093/jas/sky380 30299469 PMC6276549

[171] Otten W Kanitz E Tuchscherer M Schneider F Brussow KP. Effects of adrenocorticotropin stimulation on cortisol dynamics of pregnant gilts and their fetuses: implications for prenatal stress studies. Theriogenology. (2004) 61:1649–59. 10.1016/j.theriogenology.2003.09.009 15019461

[172] Merlot E Quesnel H Prunier A. Prenatal stress, immunity and neonatal health in farm animal species. Animal. (2013) 7:2016–25. 10.1017/S175173111300147X 23915487

[173] Rault JL Mack LA Carter CS Garner JP Marchant-Forde JN Richert BT. Prenatal stress puzzle, the oxytocin piece: prenatal stress alters the behaviour and autonomic regulation in piglets, insights from oxytocin. Appl Anim Behav Sci. (2013) 148:99–107. 10.1016/j.applanim.2013.07.001

[174] Jarvis S Moinard C Robson SK Baxter E Ormandy E Douglas AJ. Programming the offspring of the pig by prenatal social stress: neuroendocrine activity and behaviour. Horm Behav. (2006) 49:68–80. 10.1016/j.yhbeh.2005.05.004 15961089

[175] Rutherford KMD Piastowska-Ciesielska A Donald RD Robson SK Ison SH Jarvis S. Prenatal stress produces anxiety prone female offspring and impaired maternal behaviour in the domestic pig. Physiol Behav. (2014) 129:255–64. 10.1016/j.physbeh.2014.02.052 24631303

[176] Rutherford KM Robson SK Donald RD Jarvis S Sandercock DA Scott EM. Pre-natal stress amplifies the immediate behavioural responses to acute pain in piglets. Biol Lett. (2009) 5:452–4. 10.1098/rsbl.2009.0175 19411272 PMC2781921

[177] Brajon S Ringgenberg N Torrey S Bergeron R Devillers N. Impact of prenatal stress and environmental enrichment prior to weaning on activity and social behaviour of piglets (Sus Scrofa). Appl Anim Behav Sci. (2017) 197:15–23. 10.1016/j.applanim.2017.09.005

[178] Held SD Špinka M. Animal play and animal welfare. Anim Behav. (2011) 81:891–9. 10.1016/j.anbehav.2011.01.007

[179] Kranendonk G Hopster H van Eerdenburg F van Reenen K Fillerup M de Groot J. Evaluation of oral administration of cortisol as a model for prenatal stress in pregnant sows. Am J Vet Res. (2005) 66:1419. 10.2460/ajvr.2005.66.780 15934605

[180] Lay DC Kattesh HG Cunnick JE Daniels MJ Kranendonk G McMunn KA. Effect of prenatal stress on subsequent response to mixing stress and a lipopolysaccharide challenge in pigs. J Anim Sci. (2011) 89:1787–94. 10.2527/jas.2010-3612 21606444

[181] Merlot E Pastorelli H Prunier A Pere MC Louveau I Lefaucheur L. Sow environment during gestation: part I. Influence on maternal physiology and lacteal secretions in relation with neonatal survival. Animal. (2019) 13:1432–9. 10.1017/S1751731118002987 30468144

[182] Quesnel H Peuteman B Pere MC Louveau I Lefaucheur L Perruchot MH. Effect of environmental enrichment with wood materials and straw pellets on the metabolic status of sows during gestation. Livest Sci. (2019) 229:43–8. 10.1016/j.livsci.2019.09.005

[183] Quesnel H Pere MC Louveau I Lefaucheur L Perruchot MH Prunier A. Sow environment during gestation: part II. influence on piglet physiology and tissue maturity at birth. Animal. (2019) 13:1440–7. 10.1017/S1751731118003087 30442216

[184] Tatemoto P Bernardino T Alves L Souza ACD Palme R Zanella AJ. Environmental enrichment for pregnant sows modulates hpa-axis and behavior in the offspring. Appl Anim Behav Sci. (2019) 220:104854. 10.1016/j.applanim.2019.104854

[185] Parada Sarmiento M Bernardino T Tatemoto P Polo G Zanella AJ. Your pain is not my gain: how the in-utero experience of piglets born from sows with lameness shapes their life trajectory. Sci Rep. (2021) 11:13052. 10.1038/s41598-021-92507-2 34158529 PMC8219680

[186] Sarmiento MP Thiago B Patricia T Adroaldo Z. Agression, vocalization and underweight in piglets born from gilts with lameness. In: Proceedings of the 51st Congress of the International Society for Applied Ethology. Aarhus: ISAE (2017).

[187] Bernardino T Tatemoto P Morrone B Rodrigues PHM Zanella AJ. Piglets born from sows fed high fibre diets during pregnancy are less aggressive prior to weaning. PLoS ONE. (2016) 11:e0167363. 10.1371/journal.pone.0167363 27907173 PMC5132218

[188] Johnson J Sanz Fernandez M Seibert J Ross J Lucy M Safranski T. In utero heat stress increases postnatal core body temperature in pigs. J Anim Sci. (2015) 93:4312–22. 10.2527/jas.2015-9112 26440331

[189] Safranski T Lucy M Rhoades J Estienne M Wiegert J Rhoads M. Reproductive performance of gilts having developed in heat stressed dams. J Anim Sci. (2015) 93:85.

[190] Proctor J Lugar D Lucy M Safranski T Stewart K. Effects of in utero heat stress on boar growth and reproduction prior to, during, and after puberty. J Anim Sci. (2017) 95:193. 10.2527/asasmw.2017.398

[191] Otten W Kanitz E Tuchscherer M. Prenatal stress in pigs: effects on growth, physiological stress reactions and immune function. Archiv Fur Tierzucht. (2000) 43:159–64. 19350805

[192] Xu J Li Y Yang Z Li C Liang H Wu Z. Yeast probiotics shape the gut microbiome and improve the health of early-weaned piglets. Front Microbiol. (2018) 9:2011. 10.3389/fmicb.2018.02011 30210480 PMC6119770

[193] Campos PHRF Silva BAN Donzele JL Oliveira RFM Knol EF. Effects of sow nutrition during gestation on within-litter birth weight variation: a review. Animal. (2012) 6:797–806. 10.1017/S1751731111002242 22558927

[194] Tuyttens FAM. The importance of straw for pig and cattle welfare: a review. Appl Anim Behav Sci. (2005) 92:261–82. 10.1016/j.applanim.2005.05.007

[195] Stewart CL O'Connell NE Boyle L. Influence of access to straw provided in racks on the welfare of sows in large dynamic groups. Appl Anim Behav Sci. (2008) 112:235–47. 10.1016/j.applanim.2007.09.006

